# Supplementary material for: Inhibitory Effect of Hesperidin on the Expression of Programmed Death Ligand (PD-L1) in Breast Cancer
Source: Molecules. 2020 Jan 8;25(2):252. doi: 10.3390/molecules25020252 (PMC7024188; doi:10.3390/molecules25020252)
Supplement: Supplementary file 1 [file molecules-25-00252-s001.pdf]

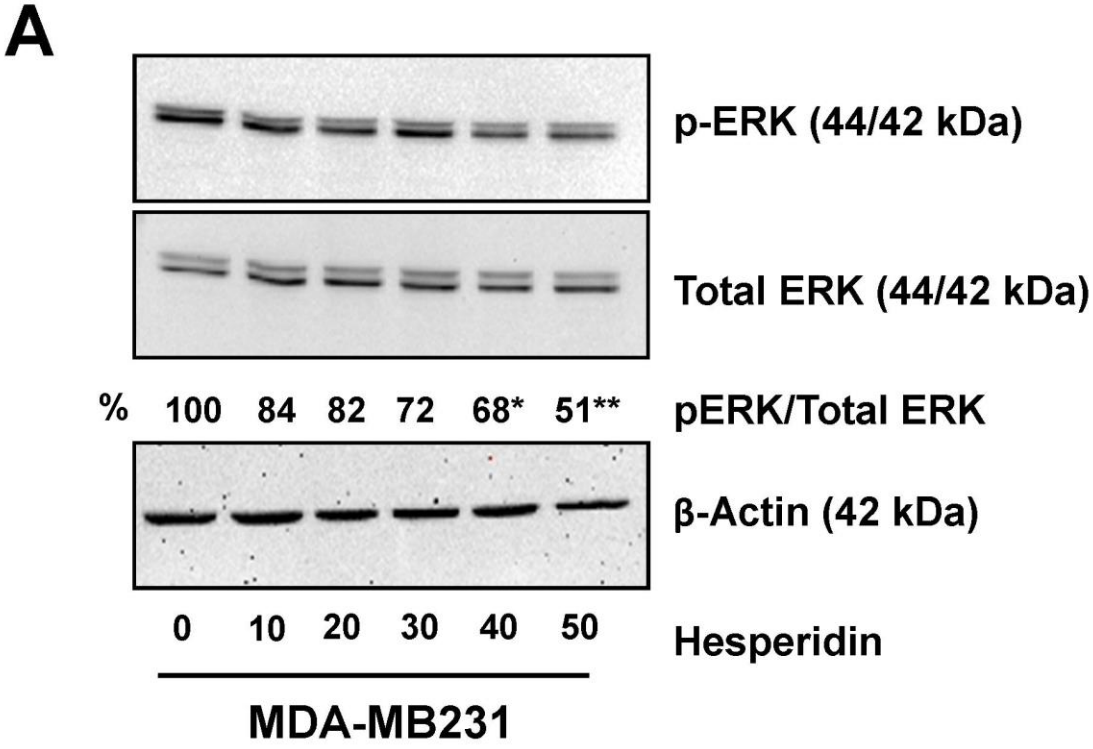

Figure S1. Downregulation of ERK phosphorylation in MDA-MB231 cells treated with hesperidin **(A)** phosphorylation of ERK

Table S1. The half of effective concentration (EC_{50}) from mRNA level, protein level and MMP secretion measured by Realtime PCR, Western blot and Gelatin zymography expressed as mean± SD (n = 3, each)

Experiment targets	EC50
PD-L1 expression	
mRNA level	38.48±8.13 μM
Protein level	41.38±3.90 μM
Signaling expression	
Akt expression	30.56±8.56 μM
NF-κB expression	38.90±6.79 μM
MMP Secretion	
MMP-9	42.14±3.23 μM
MMP-2	12.17±1.57 μM
